# Nutritive Value of Foods

**Published:** 1891-03

**Authors:** 


### COMPARATIVE NUTRITIVE VALUE OF FOODS.

By taking for basis the tables of Pavy, Letheby, Smith, and other recognized authorities on food, and comparing the amount of salts with the total nutritive value of various food substances, the following will be found to be the percentage of each one of the several classes.

GRAINS.

| | | |
|---|---|---|
| Bread, | 2. 7 | per cent. |
| Wheat, | 2. | " |
| Oatmeal, | 3. 5 | " |
| Indian Meal, | 2. | " |
| Rice, | . 6 | " |
| Average, | 2. 16 | " |

ANIMAL FOOD.

| | | |
|---|---|---|
| Milk, | 5. 7 | per cent. |
| Eggs, | 5. 8 | " |
| Cheese, | 8. | " |
| Lean Beef, | 18. 2 | " |
| Average, | 9. 4 | " |

FRUITS.

| | | |
|---|---|---|
| Grapes, | 1. 8 | per cent. |
| Blackberries, | 3. | " |
| Cherries, | 4. 6 | " |
| Apples, | 2. 3 | " |
| Pears, | 1. 8 | " |
| Raspberries. | 3. 6 | " |
| Strawberries, | 4. 6 | " |
| Plums, | 3. 3 | " |
| Apricots, | 4. 3 | " |
| Gooseberries, | 3. 3 | " |
| Currants, | 4. 2 | " |
| Bananas, | 3. 1 | " |
| Average, | 3. 3 | " |

VEGETABLES.

| | | |
|---|---|---|
| Potatoes, | 2. 8 | per cent. |
| Turnips. | 6. 7 | " |
| Parsnips, | 5. 5 | " |
| Average, | 5. | " |

